# A Capped Peptide of the Aggregation Prone NAC 71–82 Amino Acid Stretch of α-Synuclein Folds into Soluble β-Sheet Oligomers at Low and Elevated Peptide Concentrations

**DOI:** 10.3390/ijms21051629

**Published:** 2020-02-27

**Authors:** Thomas Näsström, Jörgen Ådén, Fumina Shibata, Per Ola Andersson, Björn C.G. Karlsson

**Affiliations:** 1 Physical Pharmacy Laboratory, Linnaeus University Centre for Biomaterials Chemistry, Linnaeus University, SE-392 31 Kalmar, Sweden; fs222xy@student.lnu.se; 2 Department of Chemistry, University of Umeå, SE-901 87 Umeå, Sweden; jorgen.aden@umu.se; 3 Department of Engineering Sciences: Applied Material Science, Uppsala University, SE-751 21 Uppsala, Sweden; perola.andersson@angstrom.uu.se

**Keywords:** α-synuclein, capped NAC 71–82 peptide, soluble β-sheet oligomers, circular dichroism spectroscopy, molecular dynamics simulations, Thioflavin T fluorescence

## Abstract

Although Lewy bodies and Lewy neurites are hallmarks of Parkinson’s disease (PD) and dementia with Lewy bodies (DLB), misfolded α-synuclein oligomers are nowadays believed to be key for the development of these diseases. Attempts to target soluble misfolded species of the full-length protein have been limited so far, probably due to the fast aggregation kinetics and burial of aggregation prone segments in final cross-β-sheet fibrils. A previous characterisation study of fibrils prepared from a capped peptide of the non-amyloid β-component (NAC) 71–82 amino acid stretch of α-synuclein demonstrated an increased aggregation propensity resulting in a cross-β-structure that is also found in prion proteins. From this, it was suggested that capped NAC 71–82 peptide oligomers would provide interesting motifs with a capacity to regulate disease development. Here, we demonstrated, from a series of circular dichroism spectroscopic measurements and molecular dynamics simulations, the molecular-environment-sensitive behaviour of the capped NAC 71–82 peptide in a solution phase and the formation of β-sheet oligomeric structures in the supernatant of a fibrillisation mixture. These results highlighted the use of the capped NAC 71–82 peptide as a motif in the preparation of oligomeric β-sheet structures that potentially could be used in therapeutic strategies in the fight against progressive neurodegenerative disorders, such as PD and DLB.

## 1. Introduction

The progressive neurodegenerative disorders, Parkinson’s disease (PD) and dementia with Lewy bodies (DLB), are characterised by features of intracellular insoluble filamentous inclusions (also known as Lewy bodies, LB) of the protein α-synuclein [1], accompanied by cell death in certain parts of the brain. The physiological function of α-synuclein is still under investigation, but data suggest that it is involved in synapse vesicular cycling [2] and stabilises soluble N-ethylmaleimide-sensitive factor attachment protein receptor (SNARE) complex assembly [3]. Alpha-synuclein is classified as an intrinsically disordered protein and contains 140 amino acids (aa). The aa sequence of the protein has been divided into three domains based on its physical properties. The domain that encompasses aa residues 1–60 is known as the N-terminal domain and it is responsible for binding lipid vesicles [4,5,6,7] and Sodium dodecyl sulphate (SDS) micelles [8] due to its amphiphilic nature. Residues 61–95 are part of the non-amyloid β-component (NAC) domain and have been proved to be important during protein misfolding [9,10]. Finally, residues 96–140 belong to the C-terminal domain of α-synuclein, a domain that has been reported to diminish the aggregation-prone nature of the full-length protein relative to C-terminally truncated variants [11,12,13]. Further detailed investigations on specific aa sequences, important for filament assembly of the full-length protein, revealed that (a) the NAC 71–82 aa stretch appears to be essential and (b) studies on β-synuclein lacking the NAC 71–82 fragment did not reveal fibril formation [14]. Altogether, these results highlight the significance for further studying the NAC 71–82 aa stretch of α-synuclein in characterising mechanisms involved during protein misfolding.

While much effort has been made to characterise the LB inclusions and their role in disease pathogenesis, the correlation between LB brain deposition and cognitive impairment/parkinsonism is still under debate [15,16]. Although a mechanistic study on the influence of phosphorylation of S87 on the aggregation propensity of α-synuclein has suggested that transition from oligomers into fibrillar species contributes to pathogenesis [17], other reports propose that early pre-fibrillar soluble species (e.g., oligomers) exert more detrimental effects on the brain cells than the insoluble LB [18,19,20,21].

Therefore, various active and passive vaccination strategies in mice have been made to develop biopharmaceuticals that target different domains of monomeric and misfolded (multimeric) α-synuclein with the aim to alter cell death and the progression of the disease. Games et al. developed a passive antibody-based therapy directly targeting α-synuclein lacking the C-terminal domain, and demonstrated reduced accumulation of truncated α-synuclein in axons together with improved motor function [22]. In order to develop an active immunotherapy against PD and to reduce the risk of T-cell activation, Mandler et al. used short peptides that were designed to bind to the C-terminus domain (110–130 aa) and hence did not represent the native epitope of full-length α-synuclein [23]. The authors reported that, upon vaccination with antibodies generated from the injection of C-terminus binding peptides, the levels of α-synuclein oligomers were reduced. Other strategies have involved the development of antibodies that target early pre-fibrillar oligomeric species. Lindström and co-workers induced α-synuclein oligomers using the reactive aldehyde 4-hydroxy-2-nonenal (HNE) and demonstrated that generated monoclonal protofibril selective antibodies, based upon this motif, reduced protein pathology [24].

To date, a number of strategies have been directed towards developing α-synuclein C-terminal domain targeting antibodies; however, the development of an immune-based therapy against a target representing the NAC amino acid stretch of α-synuclein would be of high interest due to its importance in membrane binding and fibrillisation of the full-length protein. It is however pivotal that the target structure used in such a strategy would be based on a motif associated with a pathological form of the protein (i.e., soluble oligomeric structures).

We recently presented data from studies on the role of the NAC domain of α-synuclein in driving the misfolding of the protein into amyloid fibres. Fibrillisation of a capped peptide model of the NAC 71–82 aa stretch resulted in end-point fibrillar structures that shared β-sheet characteristics with those found in prion proteins [25]. Although these results provided valuable information on the role of the 71–82 aa stretch of α-synuclein in driving the aggregation of the protein into insoluble fibrillar structures, the behaviour of this amino acid stretch during pre-fibrillar conditions needs to be further investigated.

In this work, we provide evidence from using circular dichroism (CD) spectroscopy and Thioflavin T (ThT) fluorescence binding studies that a peptide representing the α-synuclein NAC 71–82 aa stretch forms soluble β-sheet oligomeric structures under pre-fibrillar conditions. These oligomeric structures could also be detected in the supernatant of a fibrillisation mixture after 72 h of incubation. SDS–peptide interaction studies revealed that the capped NAC 71–82 peptide formed β-sheet conformations at concentrations below the critical micelle concentration (CMC) of SDS. Above the CMC of SDS, the peptide was stabilised in an α-helical conformation, in agreement with that observed for the aa sequence of α-synuclein studied by nuclear magnetic resonance (NMR) spectroscopy [8]. Notably, the peptide was, after being incubated for 48 h in the absence of SDS, found to be in a random coil conformation. Molecular dynamics (MD) simulations further validated the peptide–SDS micelle interaction by revealing direct binding of both peptide models.

Our results demonstrated an environment-induced response of the secondary structure population of the capped NAC 71–82 peptide model in solution and the ability to form soluble oligomers upon prolonged incubation and at elevated peptide concentrations. Altogether, these results highlighted the use of the capped NAC 71–82 peptide in the preparation of soluble β-sheet oligomers. It can be suggested that these oligomers, when isolated, potentially could be used as target motifs in, for example, sensor diagnostics or the development of immune-based therapeutic strategies against PD and DLB.

## 2. Results and Discussion

CD spectroscopy was used to characterise the secondary structure population present in capped and non-capped NAC 71–82 peptide–SDS detergent mixtures. Studies of peptide–SDS interactions have previously been conducted to model the impact of a net negatively charged cell membrane surface on the ability of a peptide to aggregate or become embedded in the membrane [26,27,28,29]. Moreover, for α-synuclein, previous NMR spectroscopic studies have revealed coordinates for an α-helical-rich folded state on the surface of an SDS micelle [8].

**Figure 1:**
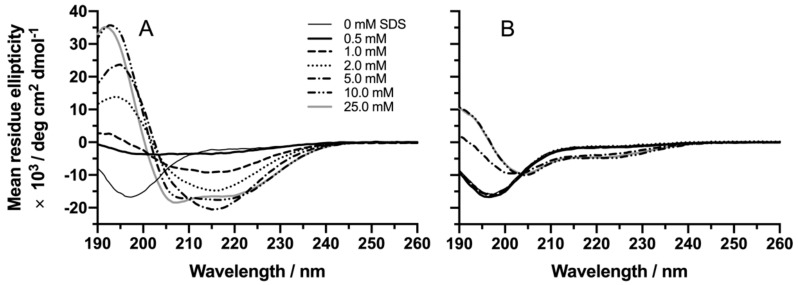
CD spectra of (**A**) 81.5 μM capped and (**B**) 85.5 μM non-capped NAC (non-amyloid β-component) 71–82 peptides with an increasing concentration (0–25 mM) of SDS in mQ water. Measured detector voltage values were never higher than 500 V for the wavelength region selected (Supplementary data, Figure S4).

CD spectroscopic titrations, after adding increasing concentrations of SDS (0–25 mM) to a constant concentration of peptide in mQ water (81.5 μM and 85.5 μM for the capped and non-capped NAC 71–82 peptide, respectively), revealed a difference in the SDS concentration-dependent folding of the peptides (Figure 1). For the capped NAC 71–82 peptide, additions of SDS in the concentration range of 0–5 mM below its CMC (8.1 mM in mQ water [30]), led to a shift in secondary structure population from a random coil conformation to a predominantly β-sheet structure (Figure 1A). This observation agreed with that observed for the amyloid-β peptide, which was found to display aggregation after being located at the particle water interface [31]. The change of secondary structure population into β-sheet formation, for the capped NAC 71–82 peptide, was based on CD spectral shifts from a minimum at 198 nm (random coil) to a single minimum at 215 nm and a maximum at 195 nm (β-sheet) in accordance with previous deconvolution of protein CD spectra [32].

Based on the observed CD spectral shifts of the capped NAC 71–82 peptide at SDS concentrations > 5 mM (at a peptide:SDS stoichiometry of ≈1:60), we concluded that the peptide, at this condition, was present in an α-helical folded state (two minima at 208 and 222 nm and a maximum at 193 nm), in line with available structural NMR data on the α-synuclein SDS micelle complex (PDB: 1XQ8) [8].

Altogether this folding behaviour of the capped NAC 71–82 peptide, at various concentrations of SDS, agrees with that reported for the full-length α-synuclein protein. Giehm et al. studied α-synuclein–SDS mixtures and demonstrated that fibrillisation of α-synuclein was dependent on the SDS/protein ratio and that the presence of SDS bulk micelles favoured α-helical folding of the protein and inhibited fibril formation [33].

Corresponding titrations of increasing concentrations of SDS to the non-capped NAC 71–82 peptide did not result in β-sheet folds at intermediate (0–5 mM) concentrations of SDS (Figure 1B). In addition, above the CMC of SDS (10 and 25 mM), spectral shifts suggested that the α-helical fold of the non-capped peptide was less favoured.

Peptide solutions, incubated up to a maximum of 48 h in the absence of SDS, were found to be present in a random coil state conformation (Supplementary data, Figure S5). Moreover, the addition of salt to a selected system (0.1 M NaCl and 10 mM SDS) had no influence on the observed secondary structure population (Supplementary data, Figures S6 and S7 for CD spectra and voltage profiles, respectively). The influence of salt on α-helical stability in the presence of SDS was as expected due to the known effect of sodium ions on reducing the repulsion of negatively charged head groups of SDS, thereby reducing CMC [30].

To conclude, it is reasonable to assume that the spectral differences observed for the studied peptides originate from the difference in terminal charges. The non-capped NAC 71–82 peptide is, based on its additional terminal charges, more water soluble than the capped NAC 71–82 peptide. This environment-sensitive behaviour of the peptide secondary structure, which was demonstrated by the capped NAC 71–82 peptide, agrees with that reported by others studying peptides related to neurodegenerative disease. Kuroda et al. studied a series of peptide fragments, derived from the prion protein (PrP) in different detergent environments, and reported a β-sheet structure propensity that was dependent upon their aa sequence [34]. A follow-up study by Di Natale and co-workers investigated the influence of another set of PrP peptide fragments and revealed that capped peptides demonstrated different α-helical/β-sheet secondary structure transitions compared to non-capped counterparts, thus providing support to the importance of hydrophobicity and peptide charge distribution for governing folding in the presence of lipid-based macromolecular targets [35].

We have recently reported that the difference in charge distribution of the capped and non-capped peptide models of the NAC 71–82 aa stretch of α-synuclein was also found to have an impact on end-point fibril morphology and structure during misfolding conditions (2 mg mL^−1^) [25]. We now provide evidence that this charge difference also has impact on the behaviour of the peptides at concentrations and conditions that do not favour aggregation (here, 0.1 mg mL^−1^).

**Figure 2:**
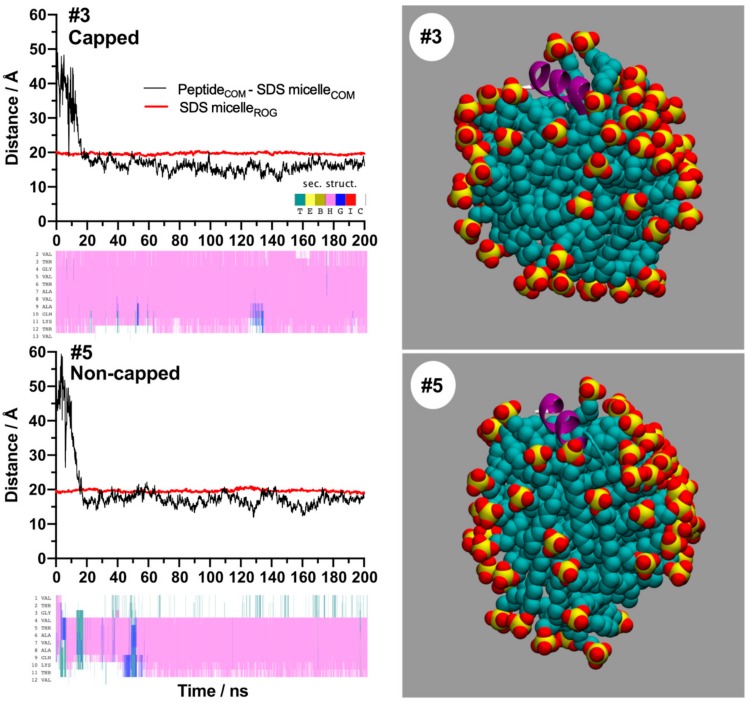
Summary of the results obtained from molecular dynamics (MD) simulations representing capped or non-capped NAC 71–82 peptide–SDS complex formation stability over time. Panels to the left depict the change in distance of the centres of masses of the peptide (Peptide_COM_) and the SDS micelle (SDS micelle_COM_) (black line), the radius of gyration of the SDS micelle (SDS micelle_ROG_, red line), and populated peptide secondary structure over time according to the STRIDE nomenclature (inserted panel: T (green) = turn, E (yellow) = extended β-sheet, B (brown) = bend, H (purple) = α-helix, G (blue) = 3_10_ helix, I (red) = π-helix, and C (white) = random coil) over time of capped (#3) and non-capped (#5) NAC 71–82 peptide representative systems. Panels to the right depict peptide–SDS complex snapshots captured after 200 ns of MD simulations.

MD simulations were conducted to add additional support to the observations made by CD spectroscopy that suggested an SDS micelle-stabilising effect on α-helical structure, which was found to be more evident for capped NAC 71–82 than the non-capped NAC 71–82 peptide. Results from MD simulations, using pre-folded α-helical starting conformations of each peptide originating from previous work [25] (Supplementary data, Figures S1 and S2) and a starting model of an SDS micelle composed of 62 detergents (resulting in a peptide:SDS stoichiometry of ≈1:62 in agreement with that observed for micelle formation after CD spectroscopic titration studies), showed that both model peptides were bound to the SDS micelle during the conditions employed (200 ns, 0.15 M NaCl). Moreover, a secondary structural analysis of MD data suggested that the capped NAC 71–82 peptide was found to be more favourably bound in an α-helical conformation than the non-capped NAC 71–82 peptide (Supplementary data, Figures S8–S15). A representative picture showing SDS binding propensities of the peptides and the involvement of a higher fraction of aas that stabilise the α-helical conformation of the capped NAC 71–82 peptide is presented in Figure 2. 

Based on the observation that the capped NAC 71–82 peptide demonstrated a molecular-environment-sensitive folding behaviour with transiently formed β-sheet folds, it was deemed important to investigate the nature of the secondary structure population of oligomers generated in the soluble fraction of a fibrillisation mixture after 72 h. Peptide fibrils were prepared and the soluble fraction was removed after centrifugation of fibrils. Compared to the other mixtures studied, the incubation of the non-capped NAC 71–82 peptide in mQ water resulted in a profound decrease in fibril formation (Supplementary data, Figure S21). Based on this observation, it was suggested that salt minimises the repulsion of terminal charges in the non-capped NAC 71–82 peptide system, which favours peptide aggregation.

**Figure 3:**
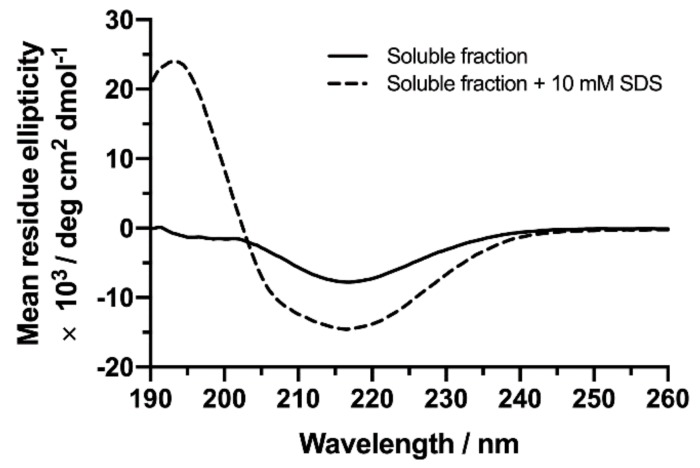
CD spectra of the soluble fraction of the capped NAC 71–82 peptide fibrillisation mixture in mQ water recorded before (total peptide concentration of 200 μM, black solid line) and after the addition of 10 mM of SDS (total peptide concentration of 160 μM, black dashed line).

A spectral analysis including peak deconvolution of recorded CD spectra, representing the soluble fraction of the capped NAC 71–82 fibrillisation composition, suggested a dominance of anti-parallel β-sheet oligomers in this sample (Figure 3, Table 1). A concentration of SDS above CMC (10 mM) was added to this fraction and a new CD spectrum was recorded. The deconvoluted peaks for the 10 mM SDS-treated soluble fraction of the capped NAC 71–82 fibrillar mixture were then inspected. A conversion from anti-parallel β-sheets (27% to 13%) to a more α-helical (10% to 32%) secondary population was observed, but coil, β-turn, and parallel β-sheet contents were less affected by the SDS treatment (Table 1). Notably, although the final peptide concentration in the supernatant of the fibrillisation mixture was in the same order of magnitude as the peptide concentration used in peptide-SDS titration experiments (0.1 mg mL^−1^ versus 0.20 mg mL^−1^ as determined by Bicinchoninic acid (BCA) analysis; Supplementary data, Figure S16) keeping the peptide:SDS stoichiometry constant at 1:60, the addition of SDS to the supernatant did not influence the secondary population (favouring α-helix formation) to the same extent as previously observed for the peptide present at lower concentrations (Figure 1).

The observation that SDS was able to favour redistribution from anti-parallel β-sheet oligomers to α-helical folds was validated using MD simulation using a starting conformation of the capped NAC 71–82 peptide in the twisted β-sheet dimer fold developed previously [25] (Supplementary data, Figure S3) and the SDS micelle model previously used in this work. Results from simulations revealed that, although the SDS micelle bound to all peptides, the starting capped NAC 71–82 anti-parallel β-sheet conformation unfolded during the performed simulations (Supplementary data, Figures S17–S20). Notably, in the sampled simulations (Supplementary data, Figure S20 #5), the SDS micelle was observed to preferentially bind monomeric forms of the peptide in α-helical conformation, again in agreement with previous experimental findings for the full-length protein. It should be added that folding of the monomers into a complete α-helical conformation was not observed during the sampled simulation time (200 ns), probably since this is a process that occurs within the μs–ms timescale. The fact that the recorded CD spectra of the fibrillisation supernatant revealed the survival of anti-parallel β-sheet oligomers motivated further characterisation of this mixture using a Thioflavin T (ThT) binding assay. ThT is a dye that has been commonly used to detect the presence of highly ordered β-sheet arrangements typically observed in amyloids [36].

**Figure 4:**
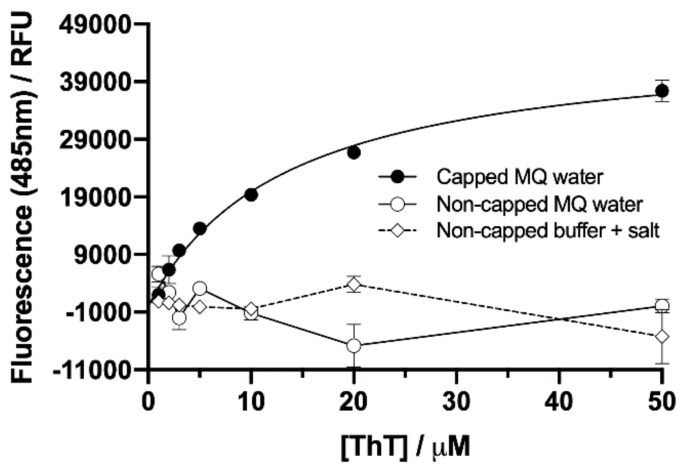
Monitored fluorescence signal at λ_em._ = 485 nm (using λ_exc._ = 445 nm) as a function of increasing ThT concentration (1–50 μM) to a constant concentration of capped (204 μM) and non-capped (213 μM) NAC 71–82 peptides from supernatants of fibrillisation mixtures prepared in mQ water (capped NAC 71–82 and non-capped NAC 71–82, closed and open circles, respectively) and Tris-HCl buffer plus 0.15 M NaCl (non-capped NAC 71–82, open diamonds). Values are presented as the mean ± the standard error of the mean from triplicate measurements. The background fluorescence signal was removed from all samples prior to analysis. A one-site-specific binding model was used to fit the data obtained for the capped NAC 71–82 peptide system (K_D_ ≈ 10 μM, R^2^ = 0.90) and the data showing the response of the non-capped NAC 71–82 peptide systems are depicted as connecting line representations.

The addition of an increasing concentration of ThT to a constant concentration of capped NAC 71–82 peptide fibrillisation mixture supernatant samples demonstrated, in contrast to that observed for the non-capped peptide NAC 71–82 in mQ water or Tris-HCl buffer and 0.15 M NaCl system, a concentration-dependent binding of ThT (Figure 4). A non-linear regression analysis of the ThT peptide-binding response of the capped NAC 71–82 peptide yielded an estimated dissociation binding constant (K_D_) of ≈10 μM. This value was comparable with that previously determined for the capped NAC 71–82 peptide bound to pre-formed fibrillar preparations including soluble species (K_D_ = 5 ± 3 μM) [25]. Altogether, these findings provided evidence of highly ordered capped NAC 71–82 β-sheet oligomers in this mixture. In addition, no fluorescence signal was observed when ThT was incubated with non-capped NAC 71–82 peptide fibrillisation mixture supernatants, hence shedding light on the inapplicability of using ThT as a reporter for fibrillar structure in this peptide system.

## 3. Materials and Methods

Chemicals: The capped NAC 71–82 aa α-synuclein peptide fragment (VTGVTAVAQKTV) (acetylated N-terminus and methyl amidated C-terminus) and the non-capped NAC 71–82 aa variant were purchased as lyophilised trifluoroacetate (TFA) salts from Caslo ApS (Kongens Lyngby, Denmark; 95.27% and 96.32% purity, respectively (HPLC and MS)). All peptide samples were purchased as pre-weighed powders (4.0 mg peptides) in Eppendorf (Hamburg, Germany) tubes. Sodium dodecyl sulphate (SDS) (≥99%, GC), Thioflavin T (ThT) (≥65% dye content, NaCl (anhydrous, ≥99%), and Tris-HCl (≥99.9%, titration) were purchased from Sigma-Aldrich (St. Louis, MO, USA). Millipore (Bedford, MA, USA) water was used for all experiments. Methanol for cleaning cuvettes was purchased from VWR Chemicals (Radnor, PA, USA).

Instruments: To facilitate peptide solvation before fibrillisation, tubes with peptides and solvent were immersed for 3 min in an ultrasound bath (2510E-DTH; Branson ultrasonics corporation, Danbury, CT, USA). Centrifugation of harvested fibrillar samples was carried out on an Eppendorf 5424 R benchtop centrifuge to isolate the soluble peptide fractions. A Tecan Spark 10M multimode plate reader (Tecan Austria GmbH, Grödig, Austria) was used for all ThT-peptide-binding fluorescence assays performed as well as for the determination of peptide concentration of the fibrillisation supernatants (as determined by a bicinchoninic acid assay, BCA). Circular dichroism (CD) analysis was carried out at 25 °C using a Jasco J-720 spectropolarimeter (Jasco corporation, Tokyo, Japan) equipped with a Peltier controller (Jasco PTC-423L, Jasco corporation) for temperature control.

Preparation of fibrils: Peptide fibrils were prepared in vitro by using a protocol previously established in our laboratory [14], and soluble fractions from aggregated preparations were isolated as follows. Buffer solution (20 mM Tris-HCl, pH 7.3 and 0.15 M NaCl) or Millipore water (mQ) was added to the pre-weighed lyophilised samples of the non-capped NAC 71–82 peptide.

Due to poor solubility of the capped NAC 71–82 peptide in isotonic solvents [25] and as determined by the supplier solubility tests, mQ water was added to the sample. The final concentration of all samples was 2 mg mL^−1^. Subsequently, all samples were treated identically using repeated steps of vortexing and ultrasound sonication. The dissolved peptide preparations were incubated in quartz Suprasil^®^ cuvettes (3.0 mL and 1 cm path length; Hellma GmbH, Müllheim, Germany) on an M22/1 magnetic stirrer at 1300 rpm with heating at 37 °C (Framo Morat GmbH, Eisenbach, Germany) for 72 h. After 72 h, fibril samples were transferred to 2 mL plastic tubes (Eppendorf, Hamburg, Germany) and centrifuged at 16,900 × *g* at 25 °C for 10 min. Resulting supernatants of both non-capped and capped NAC 71–82 fibrillisation mixtures were recovered and stored at −20 °C until further analysis.

BCA assay: Soluble fractions recovered from the in vitro fibrillisation samples of capped and non-capped NAC 71–82 preparations were measured for peptide concentration using the Pierce^®^ protein assay kit (Thermo Fisher Scientific, Waltham, MA, USA). Briefly, a working reagent was prepared using a 50:1 ratio of reagent A to B. Next, the samples and the pre-made bovine serum albumin (BSA) standards (0.025, 0.125, 0.250, 0.500, 0.750, 1.0, 1.5, and 2.0 mg mL^−1^) were diluted 1:2 in mQ water before addition into 96-well transparent Nunc F-96 MaxiSorp^®^ microplates (Thermo Fisher Scientific, Waltham, MA, USA). Finally, 25 μL of samples and standards were added in triplicates to the wells containing 200 μL of the working reagent. The plate was incubated for 30 min at 37 °C, after which it was left to cool down at room temperature for 5 min prior to absorbance measurements. The plate was assayed using a Tecan Spark 10M multimode plate reader set on a protocol as follows: ten seconds orbital shaking (amplitude = 1, frequency = 510 rpm), 5 s wait, after which absorbance was measured at 562 nm (number of flashes was set to 10 and settle time was set to 300 ms). Peptide concentrations were determined by using linear regression against known BSA protein standards in accordance with the BCA analysis method. Due to the known limitation of the BCA assay in the determination of the concentration of peptides with a variable amino acid composition and/or balance between aromatic and sulphur containing residues (Trp, Tyr, and Cys) [37,38], the non-capped NAC 71–82 peptide was used as an internal standard in the BCA assay and the results were compared to those obtained using BSA. No difference in the slopes of the generated calibration curves for the different standards used could be observed (Supplementary data, Figure S22), thus indicating the applicability of using BSA as a standard for determining the concentrations of the peptides studied in this work.

ThT binding assay: A ThT-peptide-binding assay was employed to investigate β-sheet formation in the soluble fractions recovered from the *in vitro* fibrillisation samples of capped and non-capped NAC 71–82 peptide preparations. Before addition of ThT to peptide samples, the peptide concentration of capped and non-capped NAC 71–82 soluble fractions (based upon BCA assay) was used to adjust the fractions to 0.250 mg mL^−1^ by dilution with mQ water or buffer solution, respectively. Next, all samples which were prepared in triplicates, were diluted to 1:2 in mQ water or buffer solution, and ThT was added in increasing final concentrations of either 1, 2, 3, 5, 10, 20, and 50 μM, after which the samples were transferred to fluorescence-compatible non-binding 96-well black plates (Greiner Bio-One, Kremsmünster, Austria). To control for unspecific signal due to background from either the microplates, mQ water, buffer solution or ThT, controls were made up in triplicates and readout signals were deducted from the samples. ThT fluorescence was recorded using a Tecan Spark10 M multimode plate reader set on a protocol as follows: ten seconds orbital shaking (amplitude = 1, frequency = 510 rpm), 5 s wait, followed by measurement of fluorescence intensity using an excitation wavelength of (λ_exc._) of 445 nm and an emission wavelength (λ_em._) of 485 nm (number of flashes was set to 30 and gain was set to optimal).

### 3.1. Circular Dichroism (CD) Spectroscopy

*Peptide–SDS solutions*: CD spectroscopy was used to explore secondary structure properties in freshly dissolved capped and non-capped NAC 71–82 peptide solutions. Prior to all CD measurements, the cuvette (Starna Scientific Ltd., Essex, UK) was washed with methanol, rinsed with deionised water, and dried using compressed air. Sample preparation for CD measurements was carried out as follows: 10 mL of mQ water was added to 4 mg lyophilised powder of the capped and non-capped NAC 71–82 peptides and vortexed until they were completely dissolved to reach a final concentration of 0.4 mg mL^−1^ (326 and 341 μM, respectively). From these stock solutions, 50 μL of capped and non-capped samples were diluted to a 1:4 ratio in mQ water in a CD cuvette to reach a final concentration of 0.1 mg mL^−1^ (81.5 and 85.5 μM, respectively) together with 0.0, 0.5, 1.0, 2.0, 5.0, 10, and 25 mM SDS (total volume in cuvette = 200 μL). CD spectra were recorded for each sample using 10 replicate scans employing the Jasco spectrum measurement parameters set to: sensitivity = 100 mdeg, start of scan = 260 nm, end of scan = 190 nm, data pitch = 0.5 nm, scanning mode = continuous, scanning speed = 50 nm/min, response = 2 sec, band width = 2.0 nm, and accumulation = 10 nm. For control purposes, 0.0, 0.5, 1.0, 2.0, 5.0, 10, and 25 mM SDS in mQ water background solutions were scanned and the recorded spectra were deducted from the corresponding peptide sample spectra using the Jasco spectrum analysis software v.1.53.04 (Jasco, Tokyo, Japan). For studies of secondary structure generation over time, the stock solutions (0.4 mg mL^−1^) of capped and non-capped NAC 71–82 were left for 48 h at room temperature, after which 50 μL of peptide samples were recovered every 24 h and diluted to a 1:4 ratio in mQ followed by recording of CD spectra. To ensure that the ionic strength did not influence the secondary structure of the peptides, control CD spectra of the peptides dissolved in 0.1 M NaCl solutions were also recorded.

*Analysis of the supernatant from fibrillar samples:* Soluble fractions recovered from *in vitro* fibrillisation of capped and non-capped NAC 71–82 preparations were investigated using CD spectroscopy. For this, 200 μL of a 200 μM (0.25 mg mL^−1^) fibrillar supernatant was measured 10 times and the average spectral data were presented after removing the mQ water background. In the same cuvette, 50 μL of 50 mM SDS was added, which resulted in a final concentration of 160 μM peptide (0.20 mg mL^−1^) and 10 mM SDS (total volume in cuvette = 250 μL) and a new CD spectrum was recorded.

*Deconvolution of CD spectra:* Capped and non-capped NAC 71–82 peptide–SDS samples used for CD studies were analysed for their secondary structure content using CDNN v.2.1 [39] supplemented with the Jasco software. Due to background noise in the presence of SDS at lower wavelengths, the lowest possible UV range (between 195 and 260 nm) was selected for deconvolution of secondary structure. The CDNN settings were left to default with the option “complex CD spectra” enabled, according to the user manual recommendations to obtain the highest deconvolution accuracy. Prior to analysing the CD data, the signal in mdeg was converted into mean residue ellipticity ($\theta_{MRW})$ using the equation below:

(1) $$\theta_{MRW}=\frac{\theta }{10\times N\times C\times l_{CD}}$$

where *N* is the number of peptide bonds, *C* is the molar concentration, $l_{CD}\;$ is the path length of the cuvette in cm, and θ is the observed CD signal in mdeg. Interpreted secondary structure contents in each sample are presented in percentages in Table 1.

### 3.2. Molecular Dynamics (MD) Simulations

*Peptide–SDS micelle simulations*: The coordinates for a solvated 0.15 M NaCl SDS micelle (62 lipids, 107 Na^+^ ions, 45 Cl^-^ ions, and 13,642 water molecules) were obtained from the Micelle Maker Web server (http://www.micellemaker.net) [40] using an initial model building distance of 4.0 Å between the lipids. The system size after model building was 82.33 Å × 81.97 Å × 82.09 Å.

The micellar system was initially energy-minimised to remove high-energy vdW contacts using 5000 steps of steepest descent and 5000 steps of conjugate gradient. In a second step, equilibration was performed for 100 ps at conditions of NVT (constant number of particles, volume, and temperature) keeping the SDS lipids restrained using a force constant of 10.0 kcal mol^−1^ Å^−2^. The Langevin thermostat was used with a collision frequency (gamma_ln) set to 1.0 ps^−1^ to reach the target temperature of 298.15 K. Second, 500 ps of simulation was followed at conditions of NPT (constant number of particles, pressure, and temperature) with the target pressure set to 1 bar using the Berendsen barostat and a 2 ps isotropic pressure relaxation constant. Finally, 100 ns of simulation data were collected at conditions of NPT (1 bar, 298.15 K) with no restraint on the SDS lipids.

The coordinates for the solvated micellar system obtained after 100 ns of MD simulation (final box size: 77.60 Å × 77.88 Å × 72.57 Å) were then used to build a series of peptide–SDS micellar systems. These systems were built by either adding pre-folded α-helix monomeric copies of the capped (N-terminal acetylated and C-terminal methyl amidated) or non-capped NAC 71–82 peptides or a twisted β-sheet dimer of the capped NAC 71–82 peptide to the pre-equilibrated SDS micellar system. Pre-folded structures were those previously observed from clustering of structures obtained after peptide aggregation MD simulations [25]. The peptide starting structures can be found as Supplementary data in Figures S1–S3 and the coordinates can be obtained from the authors upon request.

The peptide–SDS micellar systems were built after randomly inserting the peptides in a newly created space after elongating the *z*-axis with 20 Å with the PACKMOL software v.18.169 (Universities of Campinas and São Paulo, Brazil) [41]. As controls, simulations of solvated peptide systems without an SDS micelle (in the absence or presence of 0.15 M NaCl) were also conducted (14830 water molecules for pure water systems or 14730 water molecules and 41 Na^+^ and 41 Cl^-^ for salt solutions). In all peptide simulations, additional neutralising chloride ions were also added (one chloride ion to single monomeric pre-folded α-helix peptide structures and two chloride ions to the capped NAC 71–82 twisted β-sheet dimer system).

Simulations were thereafter performed using a similar protocol as previously described in this work; however, no restraints were used and 200 ns of simulation data were collected in the final step of the protocol. To improve statistical sampling, six replicates were setup for each peptide–SDS micellar system, with the exception of simulations of peptides in water without salt, which were only simulated as triplicates.

The time step used during MD simulations was 0.002 ps and bonds to hydrogen were constrained using the SHAKE algorithm. An 8.0 Å interaction cut-off was used for non-bonded interactions implementing periodic boundary conditions in all dimensions. The particle mesh Ewald (PME) summation method [42] was used to handle long-range electrostatic interactions, and long-range vdW interactions were treated using a continuum model correction to energy and pressure. All simulations were performed using the Amber17 software v.16 (UCSF, San Francisco, CA, USA) [43] and the GLYCAM06 (v.06j-1) [44] and Amberff14SB [45] force fields. The TIP3P water model was used and the ion parameters used were those developed by Joung and Cheatham [46].

*Analysis of MD simulation data*: The SDS micelle radius of gyration (ROG) and distances between the centre of mass (COM) of peptides and the SDS micelle were computed using the CPPTRAJ module, as implemented in AmberTools17 v.17 (UCSF, San Francisco, CA, USA) [43].

To quantify the secondary structure propensity of each non-capped and capped NAC 71–82 peptide fragment, secondary structure assignments were computed using the dictionary of secondary structure of proteins (DSSP) algorithm [47]. The total occupancy of each type of secondary structure element populated during the total MD simulation time was presented as the mean ± standard error of the mean from six separate simulations of 200 ns each. The evolution of peptide secondary structures over time was computed using the STRIDE algorithm [48] and visualised with the Timeline tool in the VMD software v.1.9.2 (University of Illinois at Urbana-Champaign, IL, USA) [49]. All snapshots from molecular simulations presented in this work were generated using VMD.

## 4. Conclusions

In this work, we investigated the folding characteristics of peptide models representing the 71–82 aa stretch of the α-synuclein protein as a means to better describe underlying factors for α-synuclein misfolding under pathological conditions. In contrast to that observed for non-capped NAC 71–82 peptide mixtures, a series of CD spectroscopic measurements performed in the absence and presence of SDS revealed the formation of β-sheet oligomers of the capped NAC 71–82 peptide at concentrations of SDS < 5 mM. At concentrations above the CMC of SDS, both the peptide model systems were found to be stabilised in an α-helical conformation. Molecular dynamics (MD) simulations were performed, which validated the binding and subsequent stabilisation of both the peptide models as α-helical conformations, although the structure of the capped NAC 71–82 peptide was observed to be more favoured in the α-helical fold than the non-capped counterpart, reflecting the role of terminal charges on folding characteristics. An analysis of the supernatant of prepared fibrillisation mixtures from the capped NAC 71–82 peptide after incubation for 72 h revealed the presence of a β-sheet structure after a ThT fluorescence binding assay. Notably, the addition of a concentration of SDS above the CMC resulted in a slight decrease of the β-sheet population, thus suggesting the survival of soluble and mature β-sheet oligomers in this system. Finally, the results presented in this work demonstrate a different environment-induced response of the secondary structure population present in the capped NAC 71–82 peptide model in relation to the non-capped counterpart, which further can be evaluated for functional effects (*e.g.*, cell viability assays) and isolated soluble β-sheet oligomers can potentially serve as target motifs for the development of therapeutic strategies and sensor-based applications.

## Figures and Tables

**Table 1:** Estimations (%) of secondary structure elements found in the supernatant of the capped NAC 71–82 peptide fibrillisation mixture in mQ water, before and after the addition of 10 mM of SDS, as determined by peak deconvolution of recorded CD spectra using the software CDNN v.2.1.

	Secondary Structure Element Populated ^a^ (%)				
System	Coil	α-Helix	β-Turn	Anti-Parallel β-Sheet	Parallel β-Sheet
Peptide/200 μM	38	10	20	27	5
Peptide/160 μM + 10 mM SDS	30	32	16	13	7

^a^ The spectral region analysed was 195–260 nm.

## Supplementary Materials

The supporting information section includes data on the systems investigated by circular dichroism spectroscopy (spectra from peptide stability measurements and spectra of secondary structure populations recorded from solutions of peptides with and without sodium chloride, and their corresponding voltage profile spectra), molecular dynamics simulations (peptide-SDS interaction stability and secondary structure propensity of the peptides upon binding to SDS), and snapshots of generated fibrils and determination of peptide concentration found in the supernatant of peptide fibrillisation mixtures using bicinchoninic acid (BCA) analysis together with the use of different standards. Supplementary Materials can be found at https://www.mdpi.com/1422-0067/21/5/1629/s1.

## Abbreviations

Aβ	Amyloid-beta
BCA	Bicinchoninic acid
BSA	Bovine serum albumin
CD	Circular dichroism
CMC	Critical micelle concentration
COM	Centre of mass
DLB	Dementia with Lewy bodies
DSSP	Dictionary of secondary structure of proteins
GC	Gas chromatography
HPLC	High-performance liquid chromatography
MD	Molecular dynamics
mQ	Millipore quality
MS	Mass spectrometry
NAC	Non-amyloid β-component
NMR	Nuclear magnetic resonance
NPT	Constant number of particles, pressure, and temperature
NVT	Constant number of particles, volume, and temperature
PME	Particle mesh Ewald
PD	Parkinson’s disease
PrP	Prion protein
ROG	Radius of gyration
SDS	Sodium dodecyl sulphate
SNARE	N-ethylmaleimide-sensitive factor attachment protein receptor
STRIDE	Structural identification
TFA	Trifluoroacetic acid
ThT	Thioflavin T
vdW	van der Waals
VMD	Visual molecular dynamics
