# Supplementary material for: A Capped Peptide of the Aggregation Prone NAC 71–82 Amino Acid Stretch of α-Synuclein Folds into Soluble β-Sheet Oligomers at Low and Elevated Peptide Concentrations
Source: Int J Mol Sci. 2020 Feb 27;21(5):1629. doi: 10.3390/ijms21051629 (PMC7084662; doi:10.3390/ijms21051629)
Supplement: Supplementary file 1 [file ijms-21-01629-s001.pdf]

Supporting information for:

A capped peptide of the aggregation prone NAC 71-82 amino acid stretch of α-synuclein folds into soluble β-sheet oligomers at low and elevated peptide concentrations

Thomas Näsström[1]*, Jörgen Ådén[2], Fumina Shibata[1], Per Ola Andersson[3], Björn C.G. Karlsson[1]*

[1]Physical Pharmacy Laboratory, Linnæus University Centre for Biomaterials Chemistry, Linnæus University, SE-392 31, Kalmar, Sweden.[2]Department of Chemistry, University of Umeå, SE-901 87, Umeå, Sweden. [3]Department of Engineering Sciences: Applied Material Science, Uppsala University, SE-751 21, Uppsala, Sweden.

Correspondence to:
* Björn C.G Karlsson
E-mail: Bjorn.karlsson@lnu.se
Tel: +46 480 446740

* Thomas Näsström
E-mail: Thomas.Nasstrom@lnu.se
Tel: +46 480 446329

Table of Contents

	Name	Page
**SELECTED PRE-FOLDED PEPTIDE STARTING STRUCTURES**		
- **α-helical capped NAC 71-82 peptide**	Figure S1	S1
- **α-helical non-capped NAC 71-82 peptide**	Figure S2	S1
- **Capped NAC 71-82 twisted β-sheet dimer**	Figure S3	S2
**CIRCULAR DICHROISM (CD) SPECTROSCOPY**		
- **SDS – peptide titration study: voltage profiles**	Figure S4	S3
- **Secondary structure stability over time**	Figure S5	S3
- **The influence of salt on secondary structure**	Figure S6	S4
- **The influence of salt on secondary structure: voltage profiles**	Figure S7	S4
**MD SIMULATIONS OF PEPTIDE-SDS INTERACTIONS**		
PEPTIDE$_{COM}$-SDS$_{COM}$ DISTANCES OVER TIME		
- **Capped NAC 71-82 α-helix monomer + an SDS micelle**	Figure S8	S5
- **Non-capped NAC 71-82 α-helix monomer + an SDS micelle**	Figure S9	S6
STRIDE SECONDARY STRUCTURE OVER TIME ANALYSIS		
- **Capped NAC 71-82 α-helix monomer + an SDS micelle**	Figure S10	S7
- **Non-capped NAC 71-82 α-helix monomer + an SDS micelle**	Figure S11	S8
DSSP SUMMARY OF SECONDARY STRUCTURE POPULATED		
- **Capped NAC 71-82 α-helix monomer +/- an SDS micelle**	Figure S12	S9
- **Non-capped NAC 71-82 α-helix monomer +/- an SDS micelle**	Figure S13	S9
SNAPSHOTS OF PEPTIDE-SDS COMPLEXES AFTER 200 ns		
- **Capped NAC 71-82 α-helix monomer + SDS micelle**	Figure S14	S10

- **Non-capped NAC 71-82 α-helix monomer + SDS micelle** Figure S15 S11

BCA ANALYSIS Figure S16 S12

MD SIMULATIONS OF THE CAPPED NAC 71-82 TWISTED β-SHEET DIMER-SDS INTERACTIONS

PEPTIDE$_{COM}$-SDS$_{COM}$ DISTANCES OVER TIME Figure S17 S13

STRIDE SECONDARY STRUCTURE OVER TIME ANALYSIS Figure S18 S14

DSSP SUMMARY OF SECONDARY STRUCTURE POPULATED Figure S19 S15

SNAPSHOTS OF PEPTIDE-SDS COMPLEXES AFTER 200 ns Figure S20 S16

SNAPSHOTS OF FIBRILLISATION MIXTURES Figure S21 S17

COMPARISON OF STANDARDS IN BCA ASSAY Figure S22 S17

SELECTED PRE-FOLDED PEPTIDE STARTING STRUCTURES

α-helical capped NAC 71-82 peptide

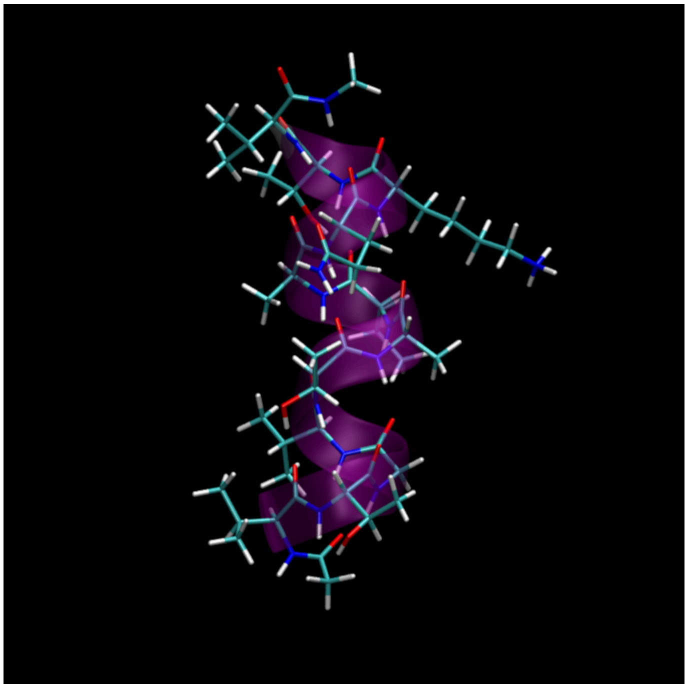

Figure S1. Capped NAC 71-82 peptide in a pre-folded α-helical structure.

α-helical non-capped NAC 71-82 peptide

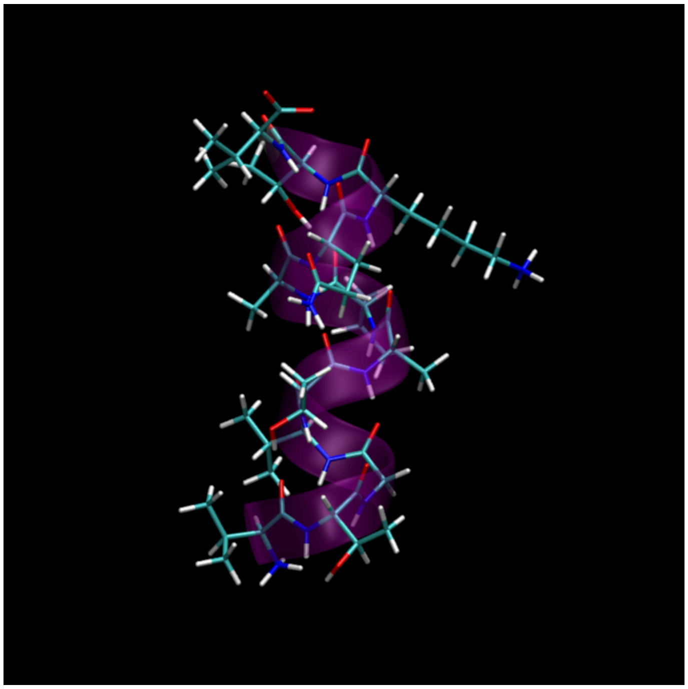

Figure S2. Non-capped NAC 71-82 peptide in a pre-folded α-helical structure.

Capped NAC 71-82 twisted β-sheet dimer

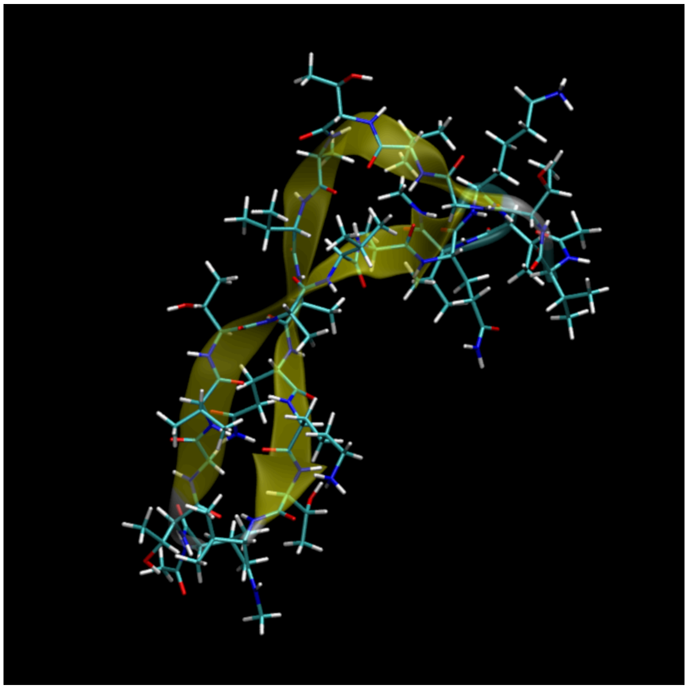

Figure S3. Capped NAC 71-82 peptide in a pre-folded twisted β-sheet dimer structure.

CIRCULAR DICHROISM (CD) SPECTROSCOPY

SDS – peptide titration study: voltage profiles

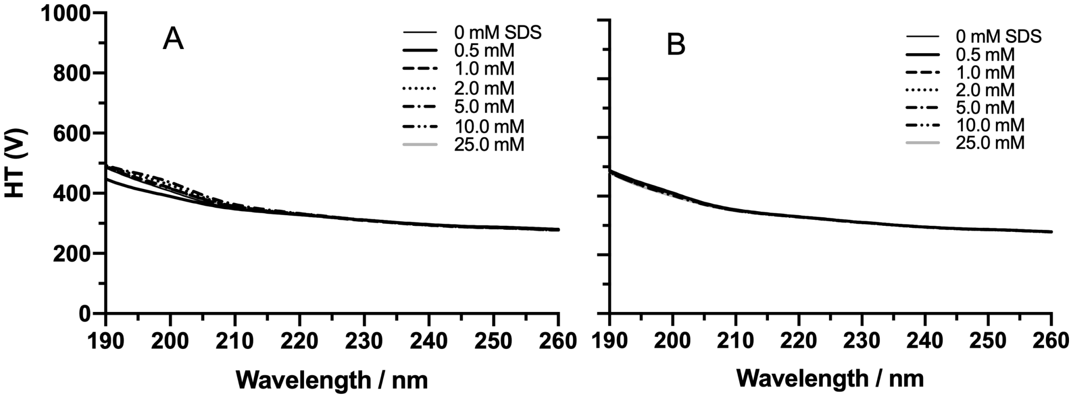

Figure S4. Circular dichroism (CD) voltage profiles of aqueous solutions with increasing concentrations of SDS (0-25 mM) of **A:** the capped (81.5 μM) or **B:** the non-capped (85.5 μM) NAC 71-82 peptide.

Secondary structure stability over time

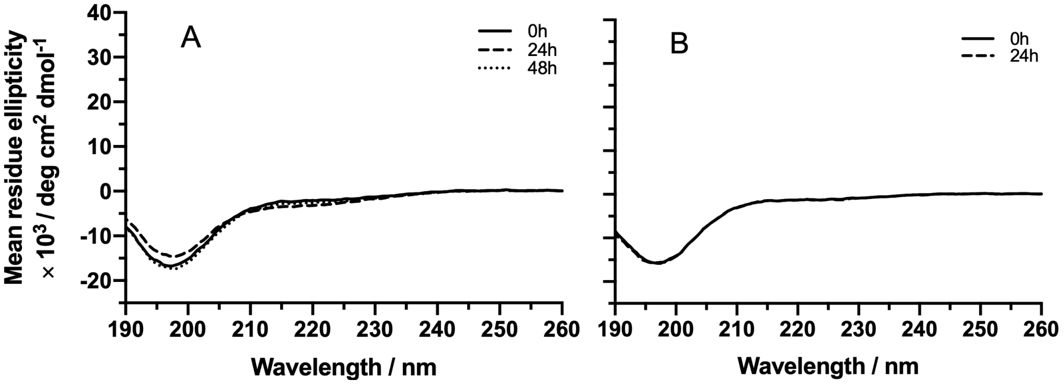

Figure S5. Circular dichroism (CD) spectra of aqueous solutions of **A:** the capped (81.5 μM) or **B:** the non-capped (85.5 μM) NAC 71-82 peptide after incubation for 0 h (solid line), 24 h (dashed line), and 48 h (dotted line).

The influence of salt on secondary structure

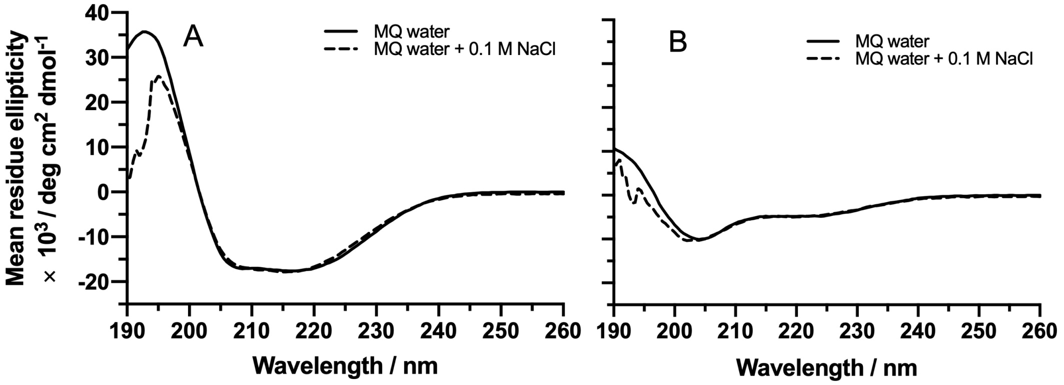

Figure S6. Circular dichroism (CD) spectra of **A:** the capped (81.5 μM) NAC 71-82 peptide in water or **B:** the non-capped (85.5 μM) NAC 71-82 peptide in water with 0.1 M NaCl in the absence (solid line) or the presence (dashed line) of 10 mM M of SDS.

The influence of salt on secondary structure: voltage profiles

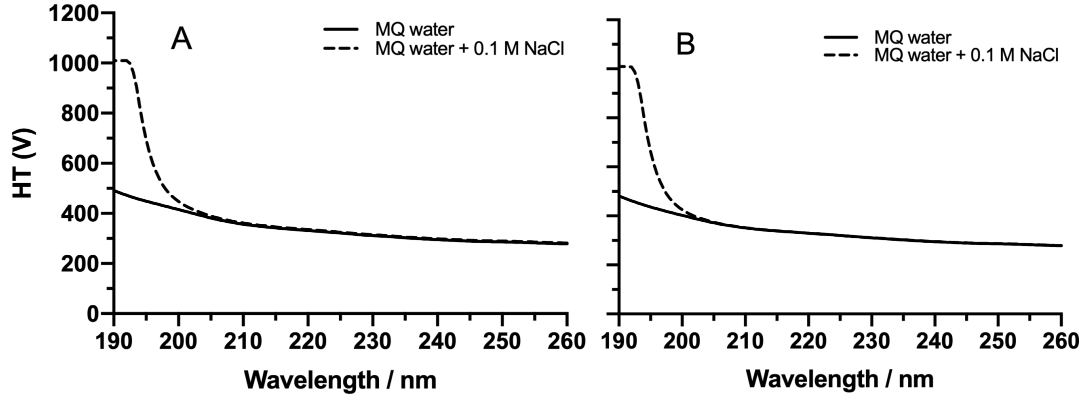

Figure S7. Circular dichroism (CD) voltage profiles of **A:** the capped (81.5 μM) NAC 71-82 peptide in water or **B:** the non-capped (85.5 μM) NAC 71-82 peptide in water and 0.1 M NaCl in the absence (solid line) or the absence (dashed line) of 10 mM of SDS.

MD SIMULATIONS OF PEPTIDE-SDS INTERACTIONS

PEPTIDE$_{COM}$-SDS$_{COM}$ DISTANCE OVER TIME

Capped NAC 71-82 α-helix monomer + an SDS micelle

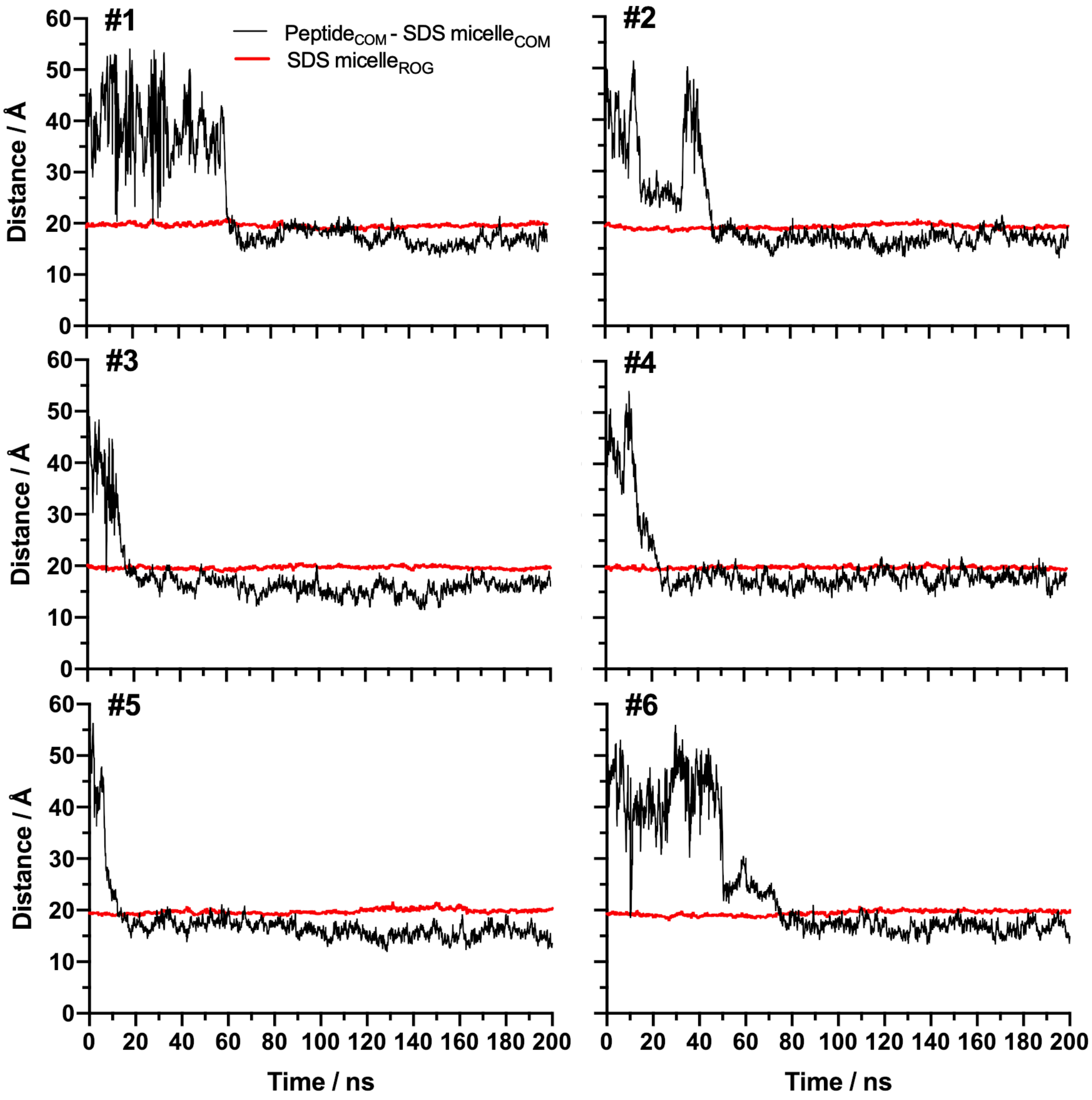

Figure S8. Capped NAC 71-82 peptide-SDS micelle centre-of-mass distances (Peptide$_{COM}$-SDS micelle$_{COM}$, black solid line) over time and the SDS micelle radius of gyration (ROG$_{SDS\ micelle}$, red line) from separate simulations (#1-#6) of 200 ns each.

Non-capped NAC 71-82 α-helix monomer + an SDS micelle

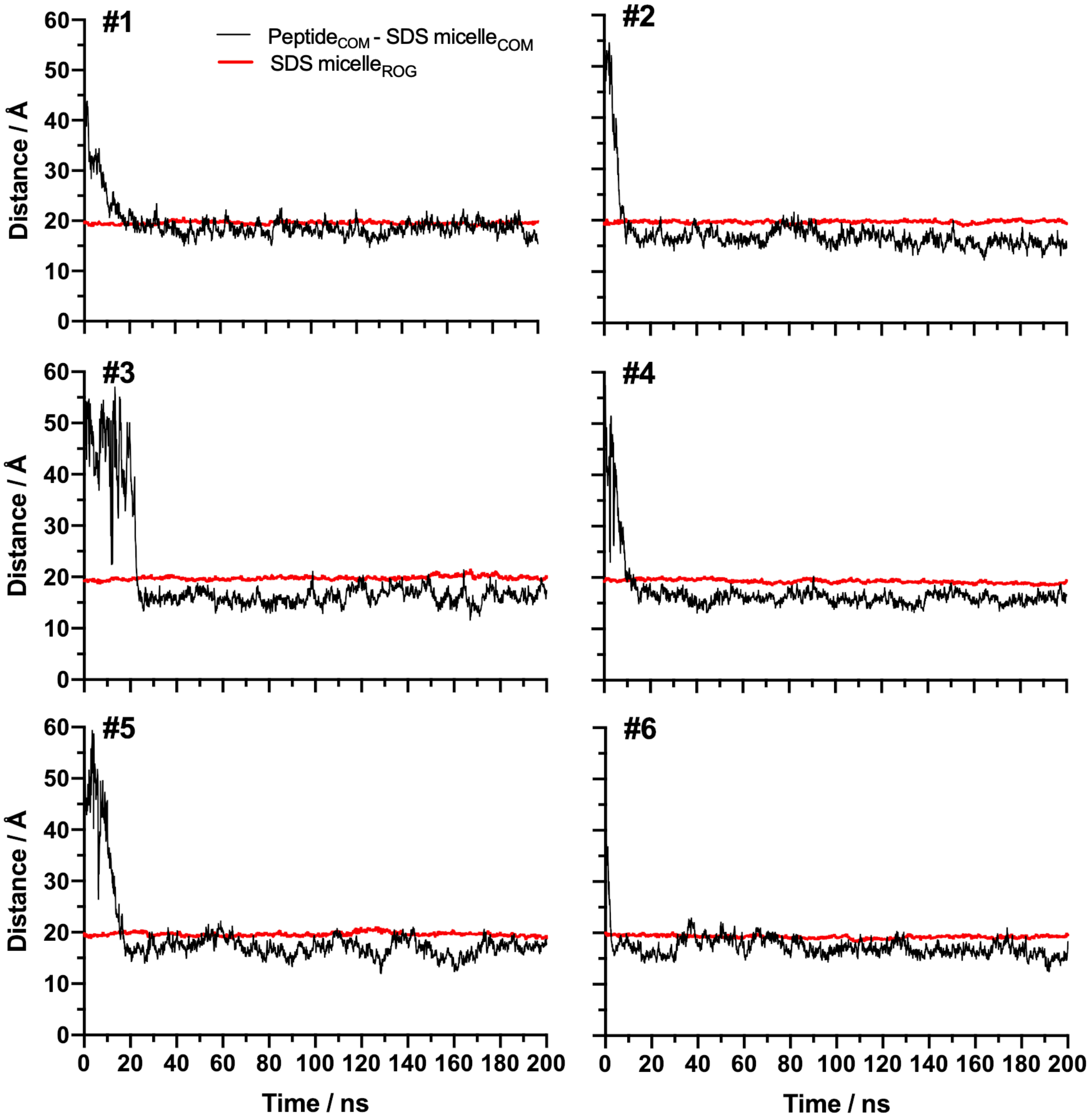

Figure S9. Non-capped NAC 71-82 peptide-SDS micelle centre-of-mass distances (Peptide$_{COM}$-SDS micelle$_{COM}$, black solid line) over time and the SDS micelle radius of gyration (ROG$_{SDS\ micelle}$, red line) from separate simulations (#1-#6) of 200 ns each.

STRIDE SECONDARY STRUCTURE OVER TIME ANALYSIS

Capped NAC 71-82 α-helix monomer + an SDS micelle

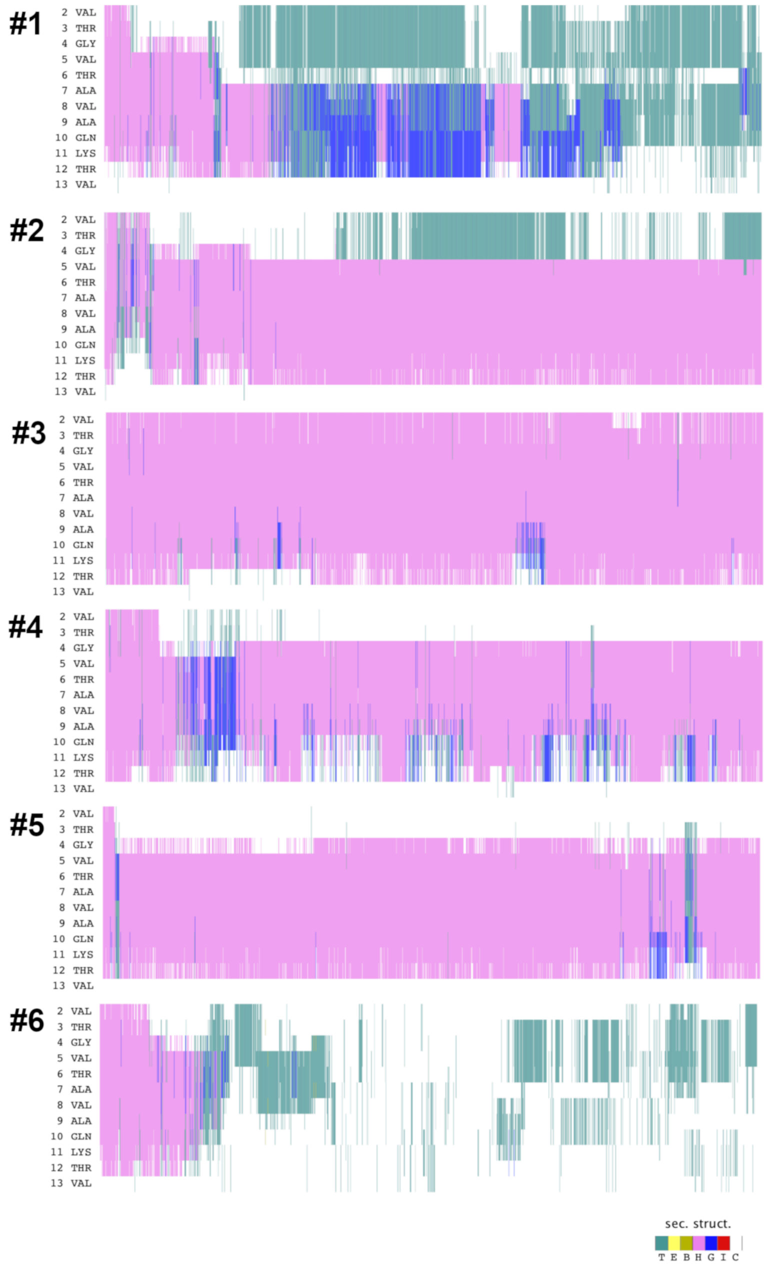

Figure S10. The evolution of secondary structure of the α-helical pre-folded capped NAC 71-82 monomer (Fig. S1) over time (200 ns) in each of totally six MD simulations (#1-#6) in the presence of an SDS micelle. The different colours shown in the inserted panel represent different secondary elements (T = turn, E = extended β-sheet, B = bend, H = α-helix, G = 3_{10}-helix, and C = random coil).

Non-capped NAC 71-82 α-helix monomer + an SDS micelle

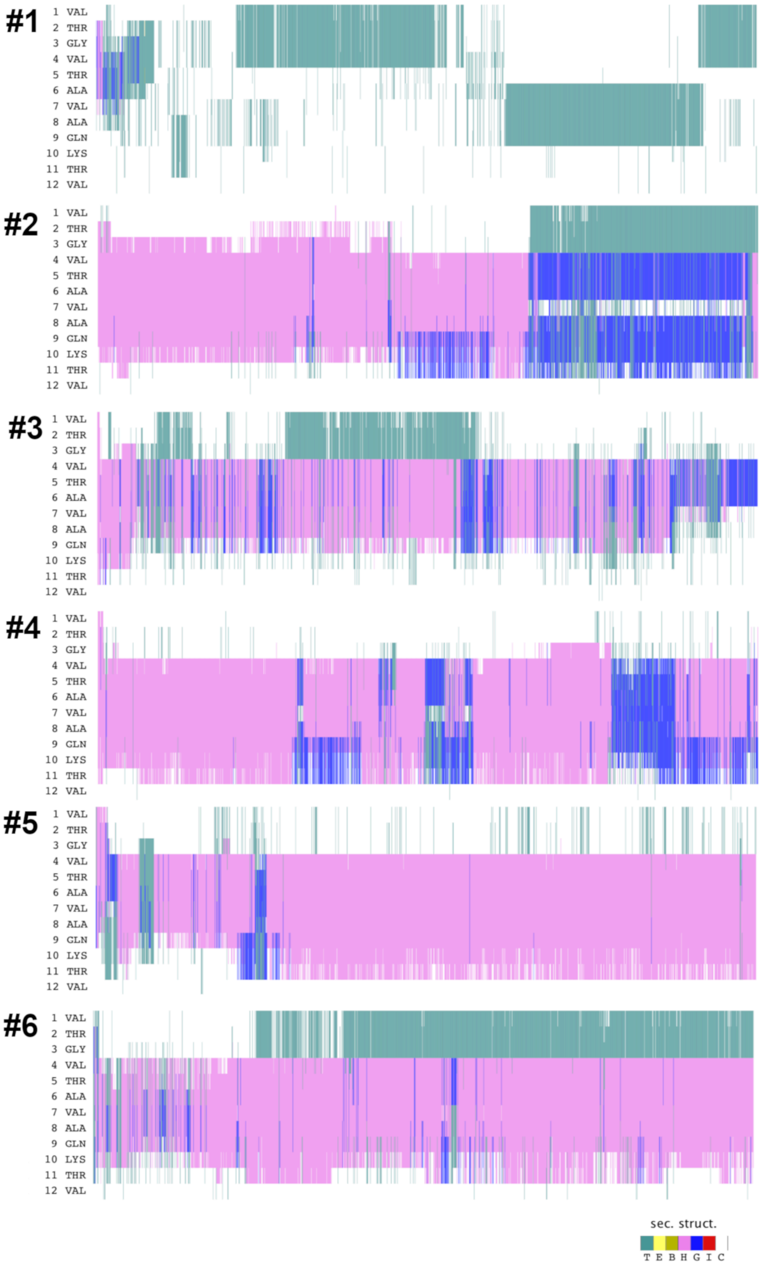

Figure S11. The evolution of secondary structure of the α-helical pre-folded non-capped NAC 71-82 monomer (Fig. S2) over time (200 ns) in each of totally six MD simulations (#1-#6) in the presence of an SDS micelle. The different colours shown in the inserted panel represent different secondary elements (T = turn, E = extended β-sheet, B = bend, H = α-helix, G = 3_{10}-helix, and C = random coil).

DSSP SUMMARY OF SECONDARY STRUCTURE POPULATED

Capped NAC 71-82 α-helix monomer +/- an SDS micelle

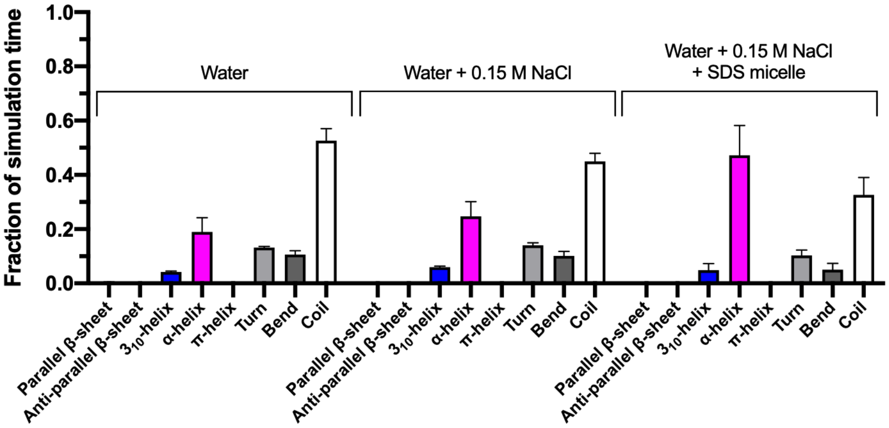

Figure S12. DSSP secondary structure analysis from MD trajectory data. Numbers describe the extent of secondary structure elements [parallel- and anti-parallel β-sheets, 3_{10}-, α-, and π-helices, hydrogen-bonded turns, bends, and no secondary structure (coil)] that were populated during MD simulations of multiple copies of the capped NAC 71-82 peptide (in explicit water (totally three simulations) and 0.15 M NaCl (totally six simulations. The total occupancy of each type of secondary structure element is presented as a fraction of the total MD simulation time, and values are presented as mean ± standard error of the mean from multiple MD simulations of 200 ns each.

Non-capped NAC 71-82 α-helix monomer in the absence and presence of an SDS micelle

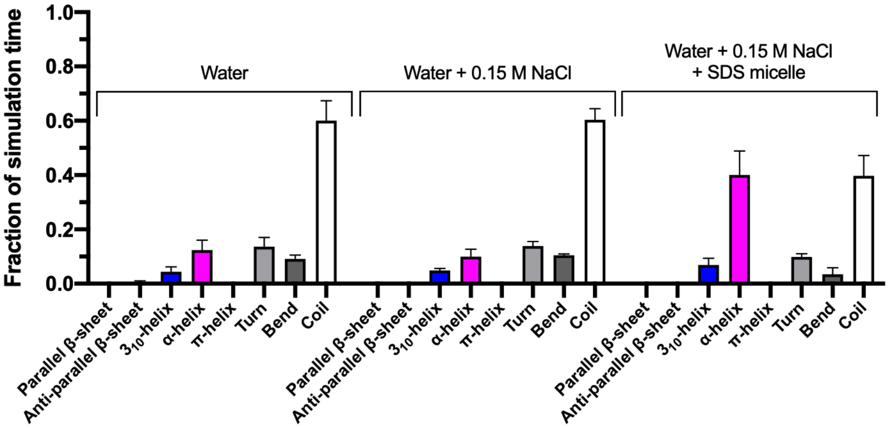

Figure S13. DSSP secondary structure analysis from MD trajectory data. Numbers describe the extent of secondary structure elements [parallel- and anti-parallel β-sheets, 3_{10}-, α-, and π-helices, hydrogen-bonded turns, bends, and no secondary structure (coil)] that were populated during MD simulations of multiple copies of the non-capped NAC 71-82 peptide (in explicit water (totally three simulations) and 0.15 M NaCl (totally six simulations. The total occupancy of each type of secondary structure element is presented as a fraction of the total MD simulation time, and values are presented as mean ± standard error of the mean from multiple MD simulations of 200 ns each.

SNAPSHOTS OF PEPTIDE-SDS COMPLEXES AFTER 200 ns

Capped NAC 71-82 α-helix monomer + an SDS micelle

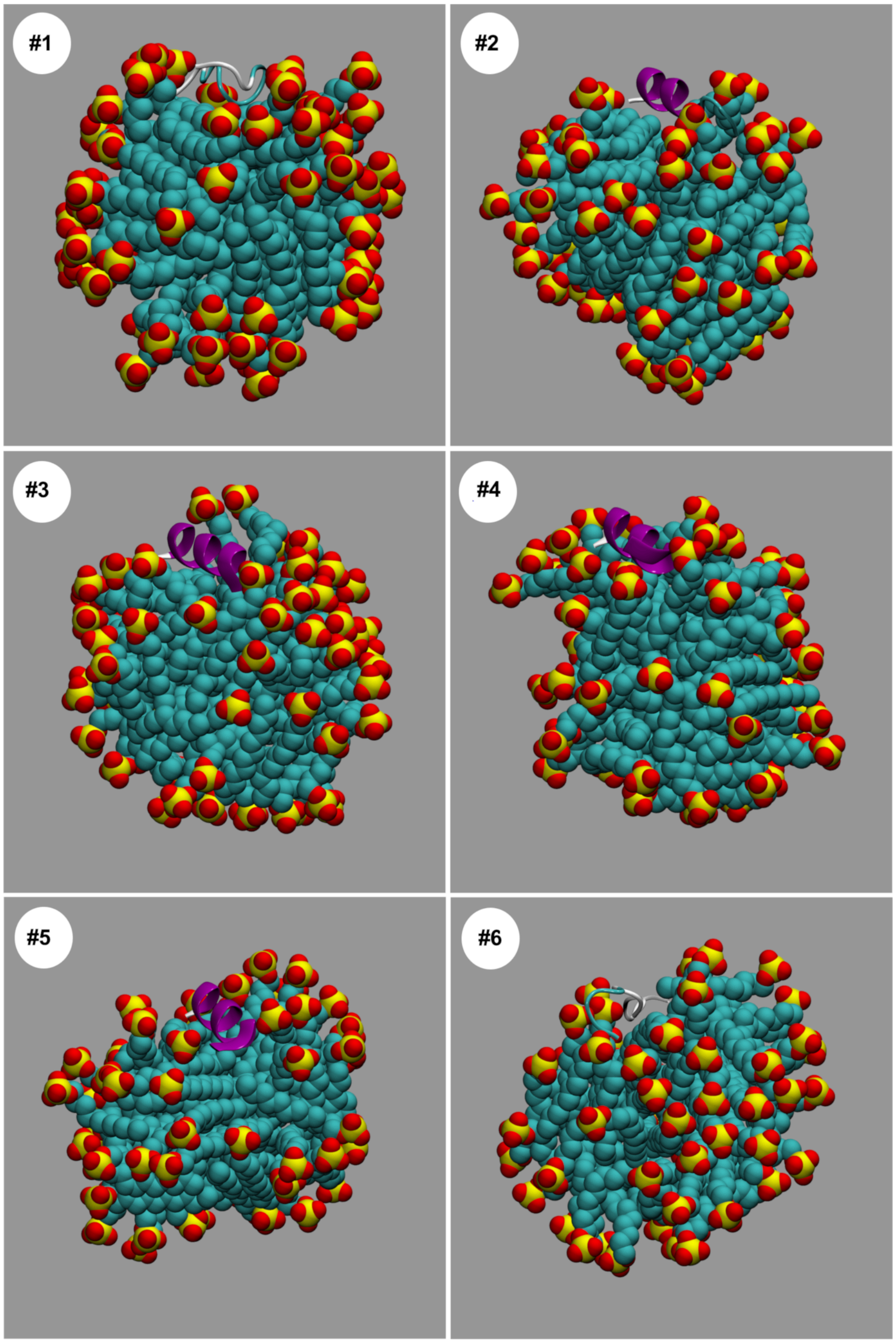

Figure S14. Snapshot of the non-capped NAC 71-82 peptide – SDS micelle complex formed after 200 ns in each of totally six MD simulations (#1-#6).

Capped NAC 71-82 α-helix monomer + an SDS micelle

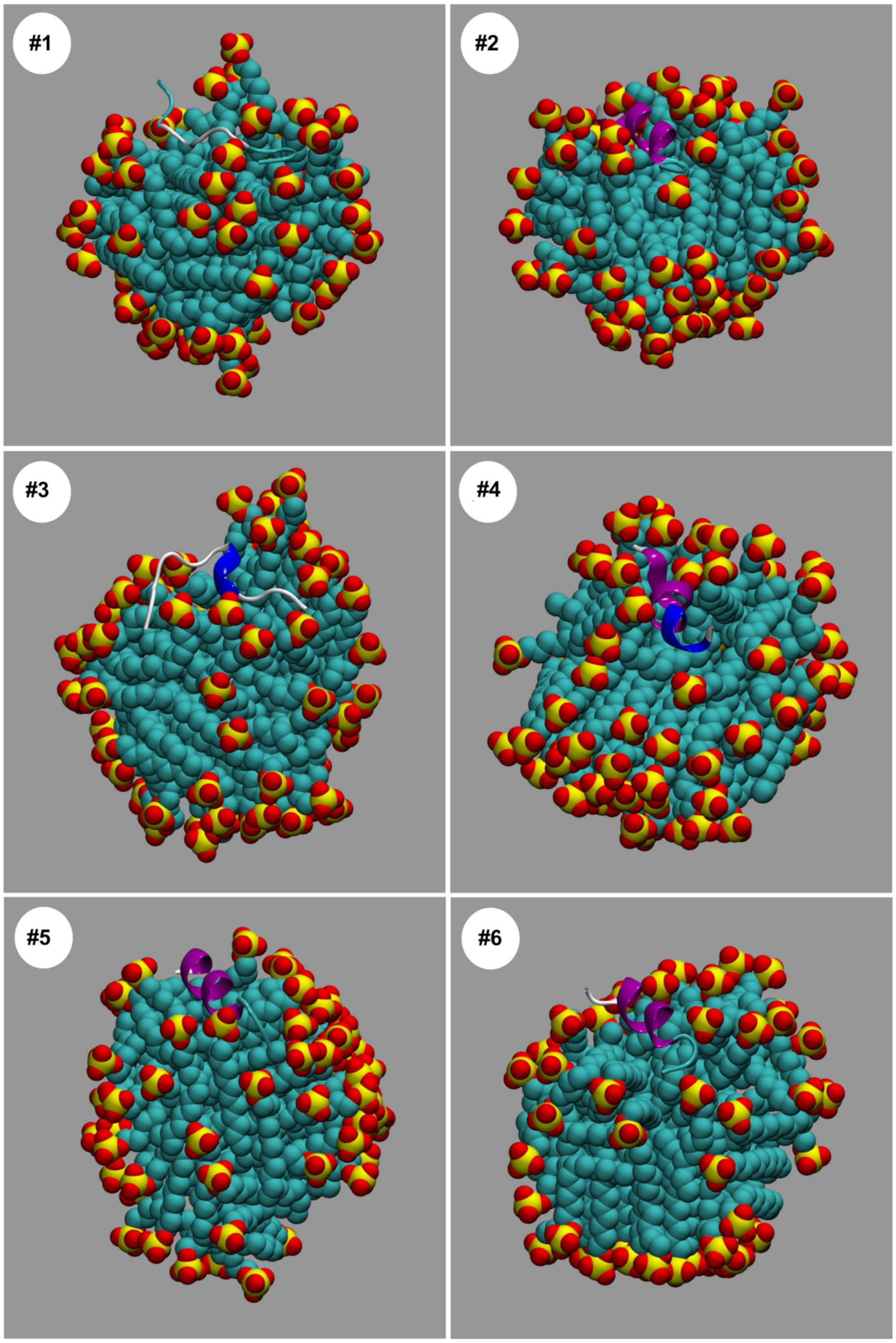

Figure S15. Snapshot of the non-capped NAC 71-82 peptide – SDS micelle complex formed after 200 ns in each of totally six MD simulations (#1-#6).

BCA ANALYSIS

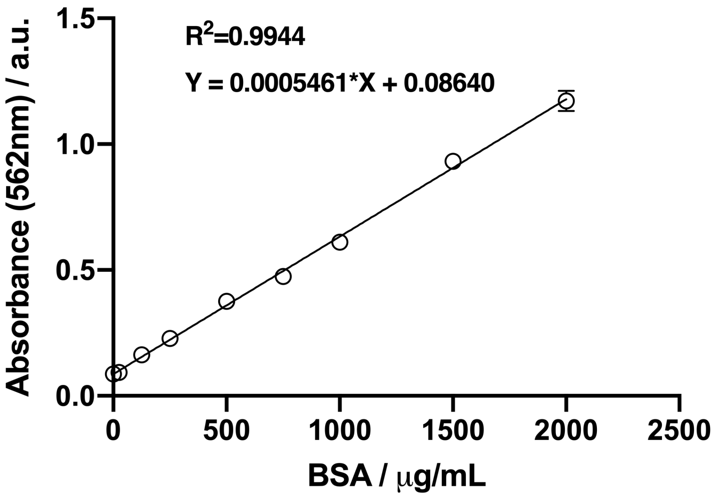

Figure S16. Data obtained after linear regression.

MD SIMULATIONS OF THE CAPPED NAC 71-82 TWISTED β-SHEET DIMER - SDS INTERACTIONS

PEPTIDE$_{COM}$-SDS$_{COM}$ DISTANCES OVER TIME

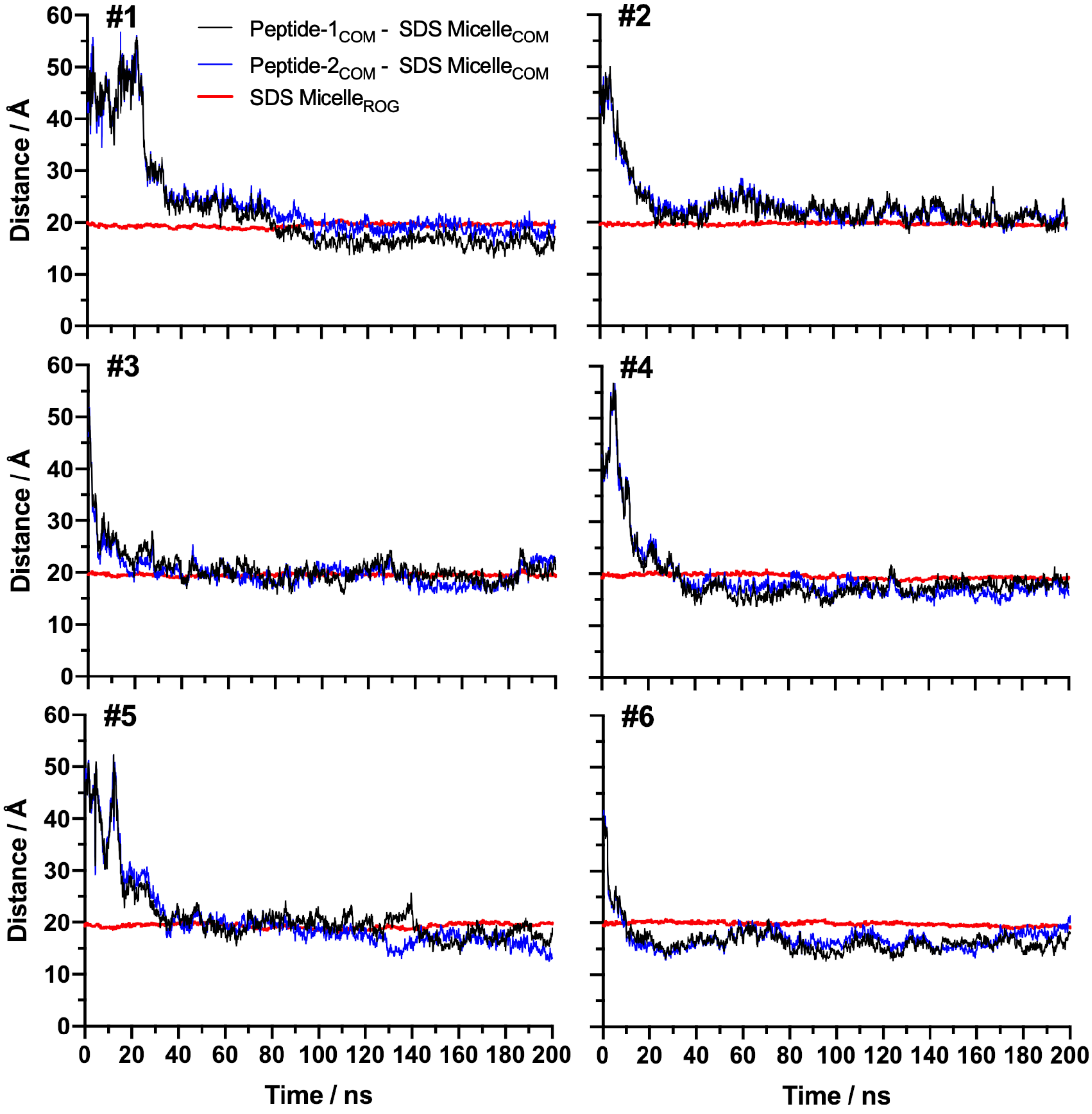

Figure S17. Capped NAC 71-82 twisted β-sheet dimer SDS micelle centre-of-mass distances (Peptide$_{COM}$-SDS micelle$_{COM}$, black and blue solid lines) over time and the SDS micelle radius of gyration (ROG$_{SDS\ micelle}$, red line) from separate simulations (#1-#6) of 200 ns each.

STRIDE SECONDARY STRUCTURE OVER TIME ANALYSIS

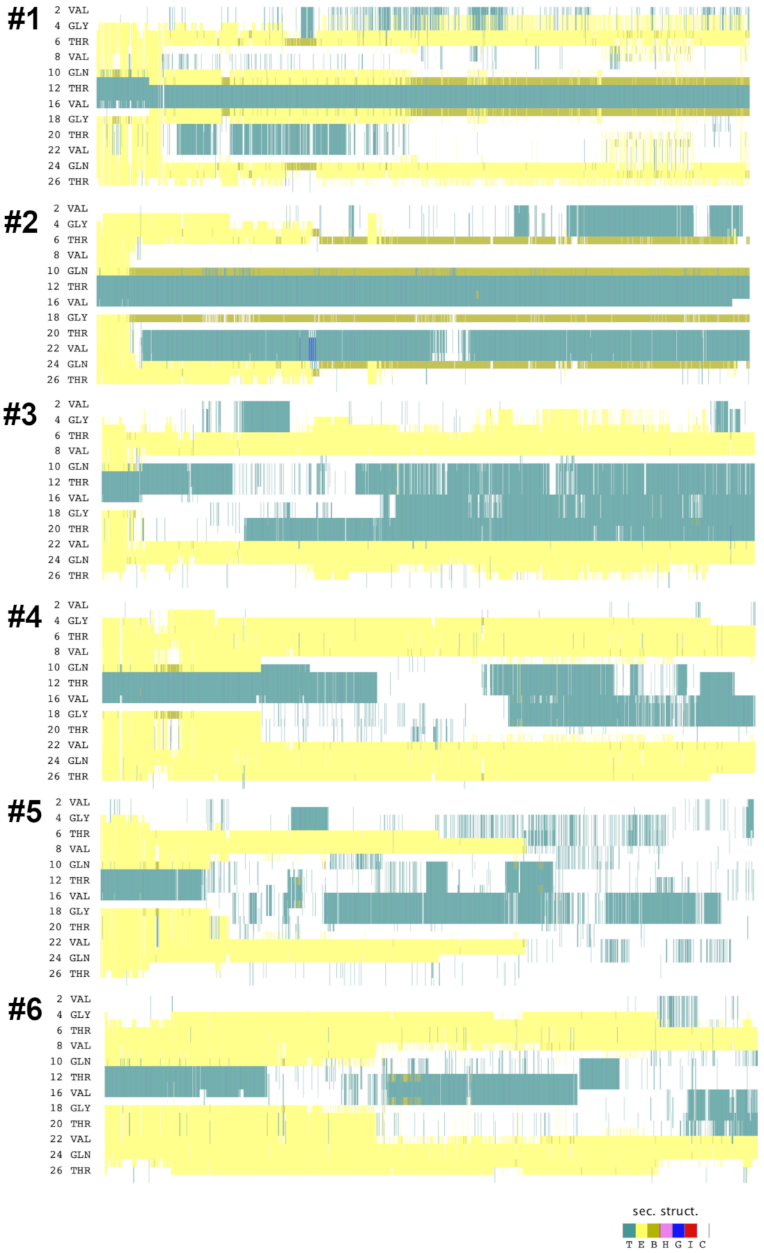

Figure S18. The evolution of secondary structure of the pre-folded twisted β-sheet capped NAC 71-82 dimer (Fig. S3) over time (200 ns) in each of totally six MD simulations (#1-#6) in the presence of an SDS micelle. The different colours shown in the inserted panel represent different secondary elements (T = turn, E = extended β-sheet, B = bend, H = α-helix, G = 3_{10}-helix, and C = random coil).

DSSP SUMMARY OF SECONDARY STRUCTURE POPULATED

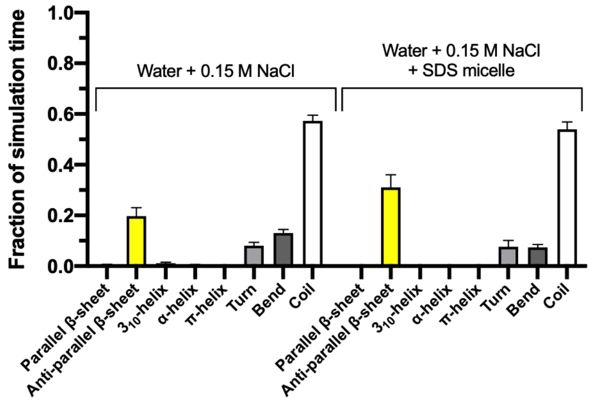

Figure S19. DSSP secondary structure analysis from MD trajectory data. Numbers describe the extent of secondary structure elements [parallel- and anti-parallel β-sheets, 3_{10}-, α-, and π-helices, hydrogen-bonded turns, bends, and no secondary structure (coil)] that were populated during MD simulations of six copies of the capped NAC 71-82 twisted β-sheet dimer in 0.15 M NaCl in the absence or presence of an SDS micelle. The total occupancy of each type of secondary structure element is presented as a fraction of the total MD simulation time, and values are presented as mean ± standard error of the mean from six MD simulations of 200 ns each.

SNAPSHOTS OF PEPTIDE-SDS COMPLEXES AFTER 200 ns

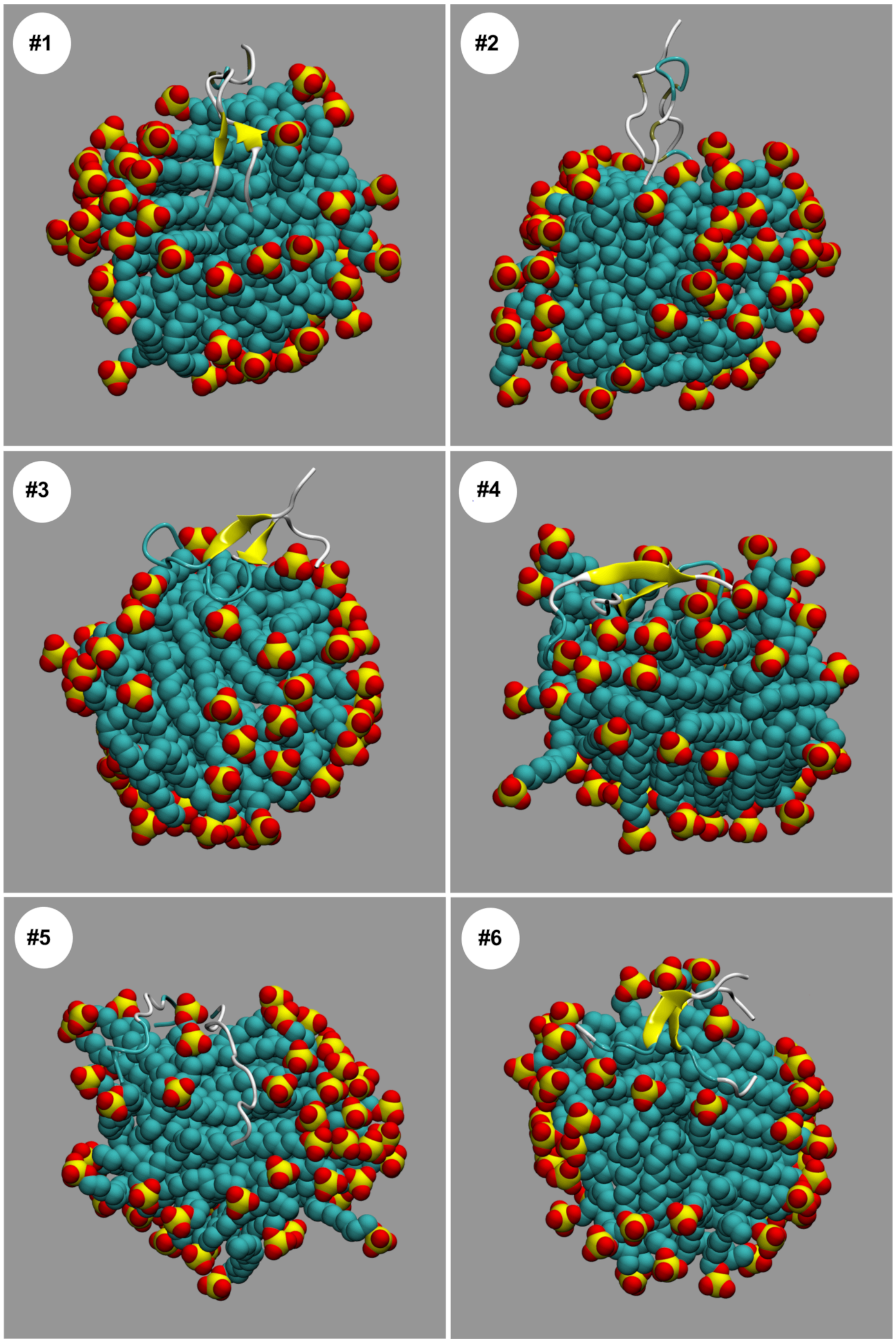

Figure S20. Snapshot of the capped NAC 71-82 twisted β-sheet dimer – SDS micelle complex formed after 200 ns in each of totally six MD simulations (#1-#6).

SNAPSHOTS OF FIBRILLISATION MIXTURES

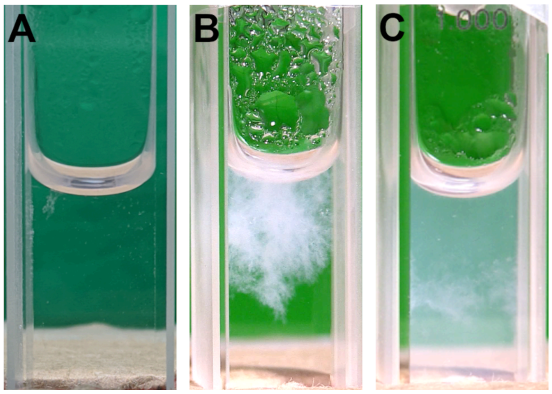

Figure S21. Fibrillisation mixture snapshots taken of **A:** the non-capped NAC 71-82 peptide in mQ water after incubation for 72 h **B:** the non-capped NAC 71-82 peptide in Tris-HCl buffer containing 0.15 M NaCl after incubation for 36 h and **C:** the capped NAC 71-82 peptide in mQ water after incubation for 60 h.

COMPARISON OF STANDARDS IN BCA ASSAY

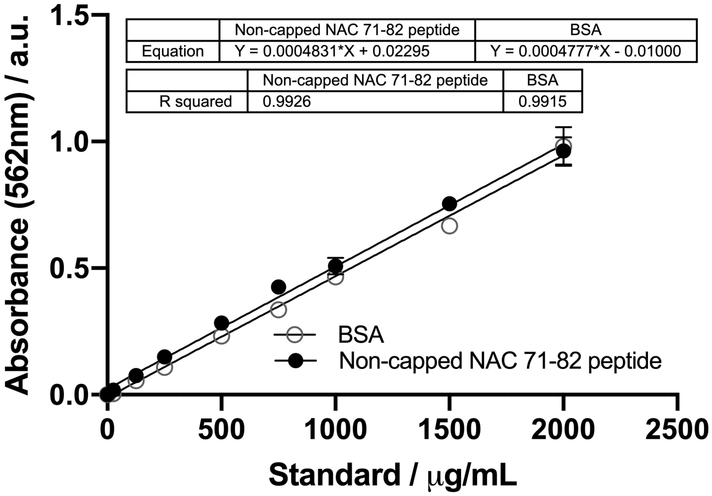

Figure S22. BCA analysis using BSA or the non-capped NAC 71-82 peptide as standard.
